# LipoDDx: a mobile application for identification of rare lipodystrophy syndromes

**DOI:** 10.1186/s13023-020-01364-1

**Published:** 2020-04-02

**Authors:** David Araújo-Vilar, Antía Fernández-Pombo, Gemma Rodríguez-Carnero, Miguel Ángel Martínez-Olmos, Ana Cantón, Rocío Villar-Taibo, Álvaro Hermida-Ameijeiras, Alicia Santamaría-Nieto, Carmen Díaz-Ortega, Carmen Martínez-Rey, Antonio Antela, Elena Losada, Andrés E. Muy-Pérez, Blanca González-Méndez, Sofía Sánchez-Iglesias

**Affiliations:** 1 Thyroid and Metabolic Diseases Unit (U.E.T.eM.), Department of Psychiatry, Radiology, Public Health, Nursing and Medicine (Medicine Area), Center for Research in Molecular Medicine and Chronic Diseases (CIMUS)-IDIS, University of Santiago de Compostela, Avda. de Barcelona 3, 15706 Santiago de Compostela, Spain; 2 Endocrinology and Nutrition Division, Complexo Hospitalario Universitario de Santiago, Santiago de Compostela, Spain; 3 Unit of Diagnosis and Treatment of Congenital Metabolic Diseases, Service of Neonatology, Department of Pediatrics, Complexo Hospitalario Universitario, CIBERER, Health Research Institute of Santiago de Compostela (IDIS), Santiago de Compostela, Spain; 4 Internal Medicine Division, Complexo Hospitalario Universitario, Santiago de Compostela, Spain; 5 Infectious Diseases Unit, Complexo Hospitalario Universitario, Santiago de Compostela, Spain; 6 Paediatrics Division. Complexo Hospitalario Universitario, Santiago de Compostela, Spain

**Keywords:** Lipodystrophy, Adipose tissue, Mobile application, Rare diseases

## Abstract

**Background:**

Lipodystrophy syndromes are a group of disorders characterized by a loss of adipose tissue once other situations of nutritional deprivation or exacerbated catabolism have been ruled out. With the exception of the HIV-associated lipodystrophy, they have a very low prevalence, which together with their large phenotypic heterogeneity makes their identification difficult, even for endocrinologists and pediatricians. This leads to significant delays in diagnosis or even to misdiagnosis.

Our group has developed an algorithm that identifies the more than 40 rare lipodystrophy subtypes described to date. This algorithm has been implemented in a free mobile application, LipoDDx®. Our aim was to establish the effectiveness of LipoDDx®.

Forty clinical records of patients with a diagnosis of certainty of most lipodystrophy subtypes were analyzed, including subjects without lipodystrophy. The medical records, blinded for diagnosis, were evaluated by 13 physicians, 1 biochemist and 1 dentist. Each evaluator first gave his/her results based on his/her own criteria. Then, a second diagnosis was given using LipoDDx®. The results were analysed based on a score table according to the complexity of each case and the prevalence of the disease.

**Results:**

LipoDDx® provides a user-friendly environment, based on usually dichotomous questions or choice of clinical signs from drop-down menus. The final result provided by this app for a particular case can be a low/high probability of suffering a particular lipodystrophy subtype. Without using LipoDDx® the success rate was 17 ± 20%, while with LipoDDx® the success rate was 79 ± 20% (*p* < 0.01).

**Conclusions:**

LipoDDx® is a free app that enables the identification of subtypes of rare lipodystrophies, which in this small cohort has around 80% effectiveness, which will be of help to doctors who are not experts in this field. However, it will be necessary to analyze more cases in order to obtain a more accurate efficiency value.

## Introduction

Lipodystrophies are a group of very heterogenous diseases characterized by a lack of adipose tissue in the absence of catabolic state or caloric deprivation [1]. According to their etiology, lipodystrophies can be congenital or acquired and, depending on the extension of the lack of fat, they can be generalized, partial or localized. On the other hand, some complex conditions such as progeria and some autoinflammatory diseases can also be associated to lipoatrophy [1, 2].

Apart from HIV-associated lipodystrophy, the remaining subtypes are extremely infrequent. The real prevalence worldwide is not known but has been estimated at around 1.3–4.7 cases per million [3]. In addition, some particular subtypes are even more infrequent with only a few cases reported so far [4–8].

These two facts, the low prevalence and the clinical heterogeneity, make difficult the diagnosis. These diseases are, therefore, not well known, even among specialists like endocrinologists or pediatricians. Although there are no specific studies about this, based on our experience with more than 200 patients evaluated in the last 15 years, the delay in diagnosis is around 20 years, with a wide range from 1 to 70.

At present, about 40 different subtypes of lipodystrophies have been described (Table 1), some of them with very similar characteristics, while others have clinical and/or biochemical, hematological, or particular image signs, although none is pathognomonic. The diagnosis of lipodystrophies is clinical [1]. On the other hand, more than 30 different genes associated with these disorders have been reported (Table 1). The identification of each subtype is critical since in more than a few cases a subtype can be related to a better or worse prognosis and/or the appearance of certain comorbidities [4, 6, 7, 9].

**Table 1:** Lipodystrophy subtypes (adapted from (2))

**1 Congenital**	
1.1 Generalized	
Type 1 CGL (*AGPAT2*, recessive, OMIM #608594)	
Type 2 CGL (*BSCL2*, recessive, OMIM #269700)	
Type 3 CGL (*CAV1*, recessive, OMIM #612526)	
Type 4 CGL (*PTRF*, recessive, OMIM #613327)	
*PPARG* -associated CGL (recessive)	
Progressive Encephalopathy with/without lipodystrophy (*BSCL2*, recessive, OMIM: #615924)	
1.2 Partial	
Type 1 FPLD (Köbberling syndrome; genes unknown, OMIM %608,600)	
Type 2 FPLD (Dunnigan disease; *LMNA*, (co-)dominant, OMIM #151660)	
Type 3 FPLD (*PPARG*, dominant, OMIM #604367)	
Type 4 FPLD (*PLIN1*, dominant, OMIM #613877)	
Type 5 FPLD (*CIDEC*, recessive, OMIM #615238)	
Type 6 FPLD (*LIPE*, recessive, OMIM #615980)	
Type 7 FPLD with congenital cataracts, and neurodegeneration (*CAV1*, dominant, OMIM #606721)	
*AKT2*-linked lipodystrophy (dominant)	
*MFN2* associated FPLD (recessive)	
*ADRA2A* associated FPLD (dominant)	
1.3 Systemic	
1.3.1 Progeroid syndromes	
Hutchinson-Gilford progeria syndrome (*LMNA*, dominant, OMIM #176670)	
Néstor-Guillermo progeria syndrome (*BANF1*, recessive, OMIM #614008)	
Atypical Werner syndrome and atypical progeroid syndrome (de novo, *LMNA*-associated)	
Werner syndrome (*RECQL2*, recessive, OMIM #277700)	
Type A mandibuloacral dysplasia (*LMNA*, recessive, OMIM #248370)	
Type B mandibuloacral dysplasia (*ZMPSTE24*, recessive, OMIM #608612)	
SHORT syndrome (*PIK3R1*, dominant, OMIM #269880)	
MDPL syndrome (*POLD1*, dominant, OMIM #615381)	
Keppen-Lubinsky syndrome (*KCNJ6*, dominant, OMIM #614098)	
Ruijs-Aalfs syndrome (*SPRTN*, recessive, OMIM #616200)	
Cockayne syndrome (*ERCC6*,recessive, OMIM #133540)	
Cockayne syndrome (*ERCC6*, recessive, OMIM #216400)	
Marfan syndrome with neonatal progeroid –like lipodystrophy (*FBN1*, dominant, OMIM #616914)	
*CAV1*-associated neonatal onset lipodystrophy syndrome (dominant)	
*PCYT1A* lipodystrophy (recessive)	
Wiedemann Rautenstrauch syndrome (*POLR3A*, recessive, OMIM #264090)	
Fontaine progeroid syndrome (*SLC25A24*, de novo, OMIM # 612289)	
1.3.2 Autoinflammatory syndromes	
PRAAS1 (*PSMB8*, recessive or digenic with *PSMA3* or *PSMB4*, OMIM #256040)	
PRAAS2 (*POMP*, dominant, OMIM #618048)	
PRAAS3 (*PSMB4*, recessive or digenic with *PSMB9*, OMIM **#** 617591)	
Panniculitis-associated lipodystrophy (*OTULIN*, recessive, OMIM #617099)	
1.3.3 Others	
Optic atrophy, cataracts, lipodystrophy/lipoatrophy, peripheral neuropathy (*OPA3*, dominant, OMIM #165300)	
**2 Acquired**	
2.1 Generalized	
Acquired Generalized Lipodystrophy, idiopathic	
Acquired Generalized Lipodystrophy, autoimmune	
Acquired Generalized Lipodystrophy, panniculitis	
2.2 Partial (excluding HIV associated lipodystrophy)	
Acquired partial lipodystrophy (Barraquer-Simons syndrome)	
Lipodystrophy associated with total body irradiation and hematopoietic stem cell transplant	
2.3 Localized	

*CGL* congenital generalized lipodystrophy, *FPLD* familial partial lipodystrophy, *PRAAS* Proteasome-associated auto-inflammatory syndrome, *MDPL* mandibular hypoplasia, deafness, progeroid features, and lipodystrophy syndrome

In order to facilitate the identification of the different lipodystrophy subtypes among non-expert doctors in this field, our group has developed an application (app) for mobile devices, based on a personal development algorithm, resulting from our experience as a national reference centre for infrequent lipodystrophies and from in-depth study of the literature. This app, called LipoDDx®, provides, with a remarkable degree of accuracy, a diagnostic approach to the suspicion of lipodystrophy in a given patient.

## Subjects and methods

The regional institutional review board, CEIC, approved this study, which was conducted according to the ethical guidelines of the Helsinki Declaration. Patients gave informed written consent for their participation in the study.

### The algorithm

**Fig. 1:**
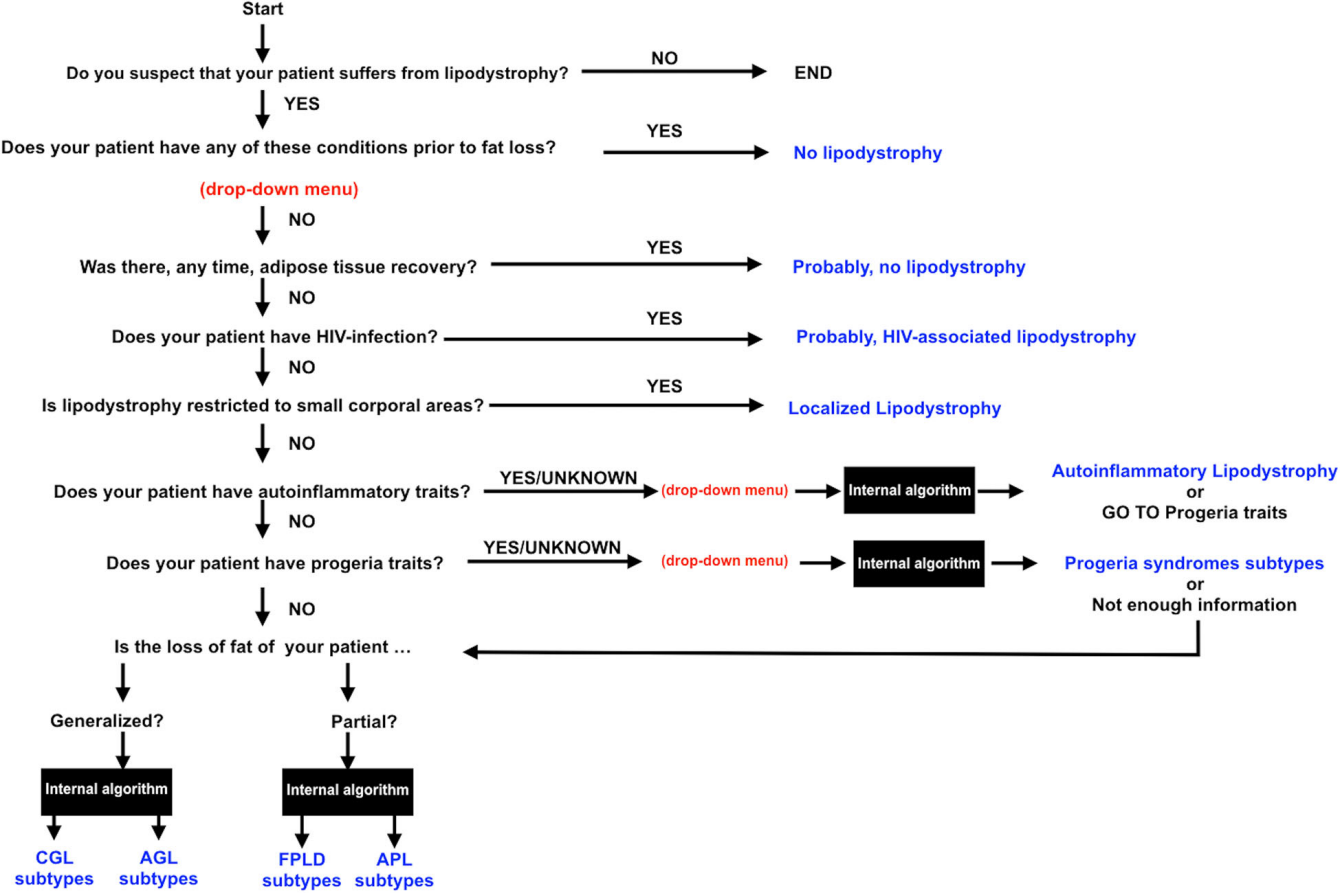
Basic algorithm of LipoDDx®. CGL: Congenital Generalized Lipodystrophy; AGL: Acquired generalized Lipodystrophy; FPLD: Familial Partial Lipodystrophy; APL: Acquired Partial Lipodystrophy

The algorithm is based on a decision tree, the basic skeleton of which is shown in Fig. 1. The detailed development of the algorithm is protected by industrial secrecy. The algorithm is presented as a decision tree based either on dichotomous questions (yes/no, yes/unknown/no) or on menus of signs/symptoms that should be chosen according to the phenotype presented by the patient. Depending on the answers that the user chooses for a specific patient, the algorithm will reach a final result either suggesting a certain subtype of lipodystrophy (indicating if the probability of suffering from that particular lipodystrophy is high or low), or stating that he/she does not suffer from lipodystrophy, or that the information provided does not enable a diagnosis.

### LipoDDx®

The software employed by the algorithm is based on the programming languages Typescript, which compiles to Javascript, HTML5 and SCSS, which compiles to CSS, allowing its installation on smartphones with IOS or Android operating systems. Other used software: Angular (version 7.x) – Licence MIT, Angular flex layout (version 7.x) – Licence MIT, Angular material (versión 7.x) – Licence MIT, ngx-translate (versión 11.x) – Licencia MIT, hammerjs (version 2.x) – Licence MIT, ng-simple-slideshow (version 1.x) – Licence MIT, Apache cordova (9.x) – Licence Apache v2.0. Project home page: https://www.uetem.com/lipoddx/. Archived version: Registro General de la Propiedad Intelectual, registration seat number 03/2019/1209.

**Fig. 2:**
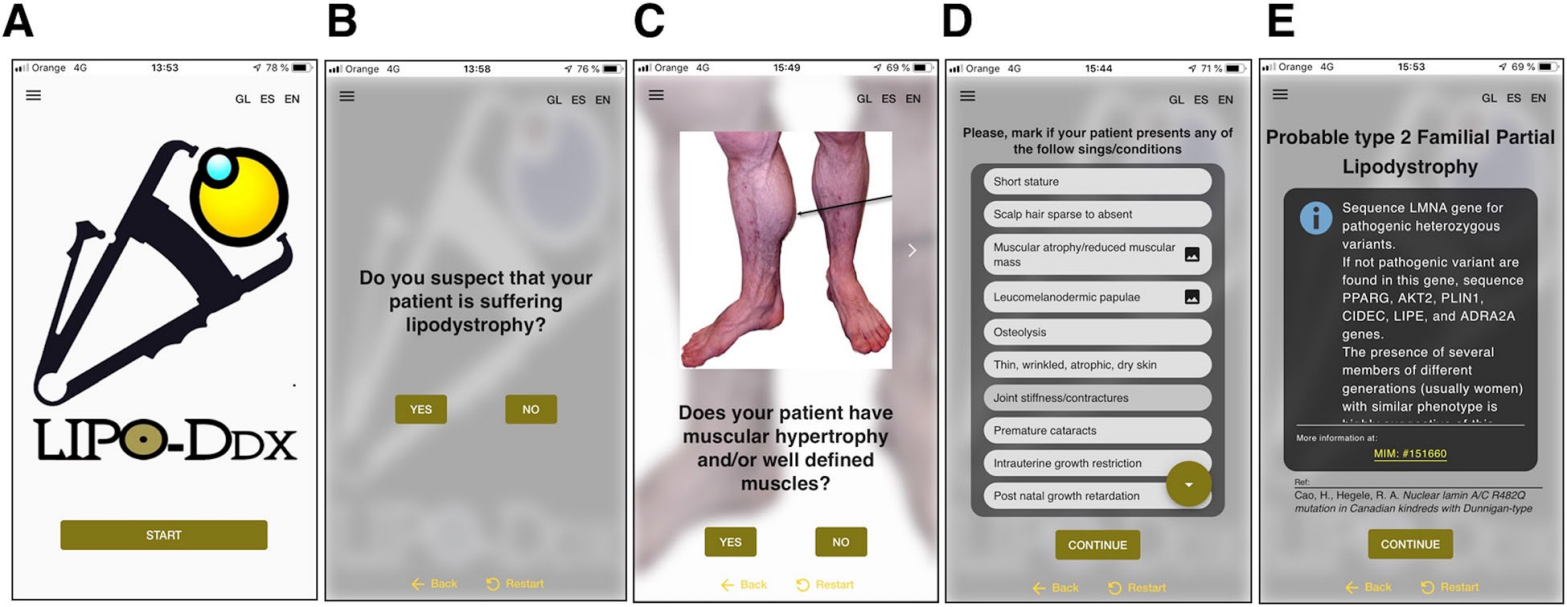
Different screens of LipoDDx®. **a** First screen. **b** Screen with dichotomous question. **c** Screen with a picture of a characteristic sign of some lipodystrophies. **d** Screen with a drop-down menu for choosing different signs. **e** Screen of a final results indicating the possible lipodystrophy subtype. In this case, information is given about the genes that should be sequenced and also links to OMIM, and bibliographic references

Like any app, LipoDDx® is based on screens showing questions about the patient’s signs or symptoms (Fig. 2). Sometimes photographs of characteristic clinical signs have been incorporated to facilitate their identification (Fig. 2c). Depending on the user’s answers, new screens will appear with further questions until a final result is reached.

On the left-hand side of the screen we have located a drop-down menu where relevant information related to intellectual property credits is provided, a disclaimer that the app can never replace the criteria of a physician, information about lipodystrophies reference centres and patient advocacy groups, as well as a link to the European lipodystrophy registry (http://134.60.15.143:8080/login.xhtml).

### Validation

To evaluate the accuracy of LipoDDx® we selected 40 clinical cases (Table 2), 19 from our unit and 21 from the scientific literature [5–7, 10–25]. Thirty-seven of them presented a diagnosis of certainty of some subtype of lipodystrophy and 3 did not present any kind of lipodystrophy. These cases, obviously without showing the diagnosis, were provided to 15 evaluators (13 physicians, specialists in endocrinology, pediatrics or internal medicine, one biochemist and one dentist). Except one, the other 14 lacked clinical experience in the management of infrequent lipodystrophies. All of them were encouraged not to study anything related to the diagnosis of lipodystrophies. Each examiner had to read each case and give their own diagnosis after which they were asked to use LipoDDx®. The results of the evaluation of the 40 cases, both those obtained based on their own knowledge and those provided by the app, were sent anonymously to a team member for further statistical analysis.

**Table 2:** Analysed cases for LipoDDX® validation

Case	Diagnosis	Reference
**#1**	Type 2 Familial Partial Lipodystrophy	Own case
**#2**	Acquired Generalized Lipodystrophy	Own case
**#3**	Acquired Partial Lipodystrophy	Own case
**#4**	Type 6 Familial Partial Lipodystrophy	Ref.# [6]
**#5**	Keppen-Lubinsky syndrome	Ref.# [10]
**#6**	Type 3 Familial Partial Lipodystrophy	Ref.# [11]
**#7**	Lipodystrophy associated with hematopoietic stem cell transplant	Own case
**#8**	Progressive Encephalopathy with/without lipodystrophy	Own case
**#9**	Type 2 congenital generalized lipodystrophy	Own case
**#10**	Type 1 congenital generalized lipodystrophy	Own case
**#11**	Marfan syndrome with neonatal progeroid –like lipodystrophy	Ref.# [12]
**#12**	Type 6 Familial Partial Lipodystrophy	Ref.# [6]
**#13**	PRAAS1	Ref.# [13]
**#14**	SHORT syndrome	Own case
**#15**	MDPL syndrome	Ref.# [14]
**#16**	Thyrotoxicosis	Own case
**#17**	Werner syndrome	Own case
**#18**	Keppen-Lubinsky syndrome	Ref.# [10]
**#19**	Type 3 Familial Partial Lipodystrophy	Ref.# [15]
**#20**	Type 4 Familial Partial Lipodystrophy	Ref.# [16]
**#22**	Anorexia nervosa	Ref.# [17]
**#23**	Localized lipodystrophy	Own case
**#24**	Cockayne syndrome	Ref.# [18]
**#25**	Type 5 Familial Partial Lipodystrophy	Ref.# [19]
**#26**	Acquired Generalized Lipodystrophy	Own case
**#27**	Type 2 Familial Partial Lipodystrophy	Own case
**#28**	*MFN2* associated FPLD	Ref.# [7]
**#29**	Type 4 congenital generalized lipodystrophy	Ref.# [20]
**#30**	Néstor-Guillermo progeria syndrome	Ref.# [21]
**#31**	Acquired Partial Lipodystrophy	Own case
**#32**	Atypical progeroid syndrome	Own case
**#33**	Mental disorder	Own case
**#34**	Hutchinson-Gilford progeria syndrome	Own case
**#35**	Type 1 congenital generalized lipodystrophy	Own case
**#36**	*ADRA2A* associated FPLD	Ref.# [5]
**#37**	Fontaine progeroid syndrome	Ref.# [22]
**#38**	Type A mandibuloacral dysplasia	Ref.# [23]
**#39**	Wiedemann Rautenstrauch syndrome	Ref.# [24]
**#40**	Type 1 Familial Partial Lipodystrophy	Own case

### Statistical analysis

Since this app is not a procedure to establish a non-disease/disease dichotomous situation, it is not possible to calculate the positive and negative predictive value. On the other hand, given the low prevalence of these disorders and the small number of cases analysed, sensitivity and specificity values ​​should not be used. In this specific case, the method used to validate our app was that of a table of variable scores (Table 1S-supplementary file), ranging between − 10 and 40 points for each case. The highest values ​​were assigned if the diagnosis was correct in the less prevalent and more complex cases, while the lowest scores were assigned to failure in the diagnosis of the simplest cases. The highest score per evaluator was 989 points, which would indicate that the diagnosis was correct in 100% of the cases. The scores were transformed into percentages and the comparison between the percentage of successes without using LipoDDx® and using LipoDDx® was performed using the Wilcoxon test. Significant differences were considered with a *p* < 0.05. All statistical analyses were performed using SPSS for Mac (release 22.0; SPSS, Chicago, IL, USA).

## Results

### App environment

LipoDDx® is an app with a user-friendly environment compatible with iOS and Android operating systems and can be downloaded for free from the Apple Store (https://apps.apple.com/es/app/lipoddx/id1474797838) and Google Play (https://play.google.com/store/apps/details?id=araujo.lipoddx). It comes in three languages ​​(English, Spanish and Galician) which can be selected in the upper right corner of the screen.

The use of the app begins with the question “do you suspect that your patient suffers from lipodystrophy?” (Fig. 2b). Depending on the response, successive screens will appear with different questions related to the patient’s symptoms and signs. Although many questions are dichotomous, sometimes the user will have to choose among a list of signs/symptoms (Fig. 2d). At the end of the process, a final screen is reached in which the lipodystrophy subtype that the patient may suffer from is suggested (Fig. 2e) or it is stated that there is not enough data to reach a diagnosis. If it is possible to identify the subtype, the app also provides information on the gene or genes that would be recommended to sequence (in the case of congenital lipodystrophies), other complementary tests that would be helpful in the diagnosis (eg. the determination of C3 complement plasma levels in Barraquer-Simons syndrome), as well as links to OMIM or ORPHANET (Fig. 2e).

### Results of the validation

**Fig. 3:**
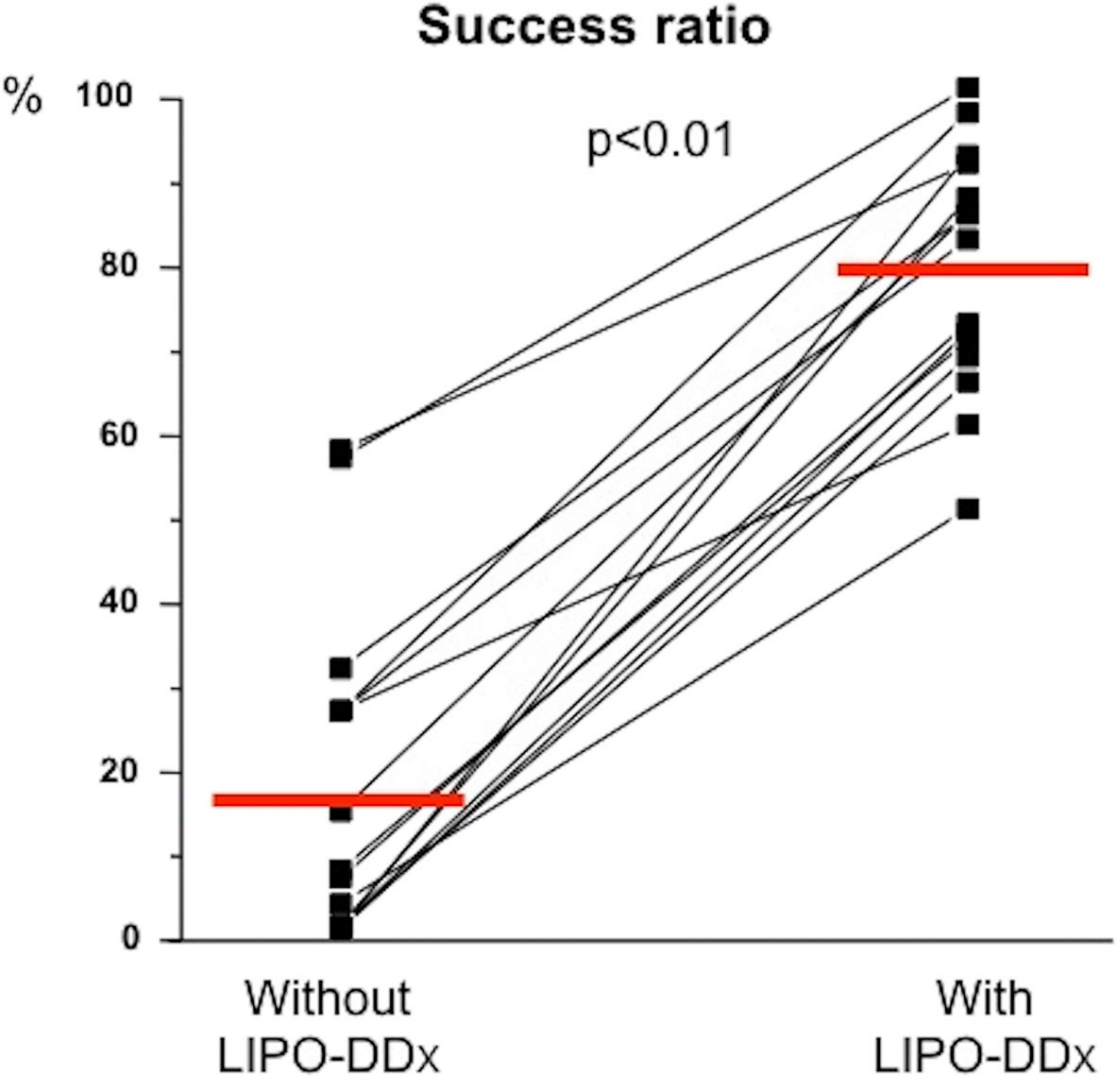
Success rate of evaluators when comparing their own diagnosis (without LipoDDx®) with the use of LipoDDx®

The results of the validation are shown in Fig. 3. Of the 40 patients analysed, the evaluators made the correct diagnosis by themselves in 17 ± 20% cases [range: 0–58%], while with LipoDDx® the success rate was 79 ± 20% [range: 60–100%] (*p* < 0.01).

## Discussion

Although without scientific evidence, the perception of experts in infrequent lipodystrophies is that there is a generalized lack of knowledge even among health-care specialists. This fact leads, as in many other rare diseases, to a significant delay in diagnosis, which in our experience is on average 20 years, mainly in partial lipodystrophies. Moreover, lipodystrophy is not a single disease but a heterogeneous set of disorders characterized by a generalized or partial absence of adipose tissue, with unique clinical manifestations, different associated co-morbidities, and a variable prognosis according to the subtype. There are few hospitals in the world with reference units specialized in lipodystrophies, and even in these units the number of patients who are diagnosed and followed do not exceed two or three hundred per centre in the best case scenario. On the other hand, the diagnosis of lipodystrophies is exclusively clinical [1], there is no complementary test or biological marker that allows for their identification.

All of this undermines the health and welfare of the patients, who are not diagnosed or, even worse, misdiagnosed, which leads to inadequate treatments and an aggravation of their complications.

Based on these premises and given the widespread use of smartphones, we have developed a mobile app, aimed at physicians, based on a personal algorithm that enables all the lipodystrophy subtypes described to date to be identified with remarkable efficiency.

The number of medical apps is huge. According to Piran et al. [26] more than 30,000 medical apps are available in Apple Store. As far as we know, LipoDDx® would be the first one that allows an accurate diagnostic approach to a large and heterogeneous group of rare diseases.

Although a success rate close to 80% can be considered a good result, we believe that this can be improved as practitioners become more familiar with the app. Furthermore, we intend to improve LipoDDx® in successive versions as new genes are discovered and new lipodystrophy subtypes are described. At the same time, the dissemination of the app in congresses and medical meetings and via social networks will probably lead to the reception of users input, which will allow us to refine the algorithm.

Realistically, it seems unlikely that any doctor will have enough experience to adequately focus the diagnosis of a certain subtype of lipodystrophy. We believe that LipoDDx® can be a useful and affordable tool for any practitioner anywhere in the world faced with a patient with suspicion of loss of adipose tissue.

As an added value, LipoDDx® provides information on expert centers in Europe and abroad that will undoubtedly serve to request second opinions or to defer patients with complex diagnoses to these centres. In addition, information is also provided on associations of patients with lipodystrophy, both European and North American. This app will therefore not only be useful to doctors but also to patients and their families.

## Conclusions

LipoDDx® is a free mobile application for the identification of different subtypes of infrequent lipodystrophies, which is effective in approximately 80% of cases in this first validation process. However, it will be necessary to analyze more cases in order to obtain a more accurate efficiency value. LipoDDx® is, to the best of our knowledge, the first app to allow a precise and rapid identification of a set of heterogenous rare diseases without having in-deep knowlegements in dysmorphology, aimed not only to specialists, but also general practitioners.

## Supplementary information

**Additional file 1: Table 1S.** Score table. The result obtained by a particular evaluator for a particular case will receive different score according to the difficulty of the diagnosis. Because the degree of difficulty in the diagnosis is not equal in all of the cases, we assigned a different score, ranged between -10 to 40, according to the “difficulty” for diagnosing. For instance, if a patient has a loss of fat because she had anorexia nervosa but the evaluator considered that she suffered any subtype of generalized lipodystrophy, the score will be negative, and the opposite is true, if a patient suffered a extremely infrequent lipodystrophy, with a complicated phenotype (for instance, Wiedemann Rautenstrauch syndrome) and the evaluator achieved a correct diagnose, the score will be high. Then, we add all the scores of each evaluator , transformed in percentage, and compared the results obtained based on his/her on knowledges with those provided by LipoDDX.

## Data Availability

LipoDDx can be download from Apple Store or Google Play. The datasets used and/or analysed during the current study are available from the corresponding author on reasonable request.

## Abbreviations

AGL: Acquired Generalized Lipodystrophy
APL: Acquired Partial Lipodystrophy
app: mobile application
CEIC: Comité de Ética de Investigación Clínica de Galicia
CGL: Congenital Generalized Lipodystrophy
CGL1: Type 1 Congenital Generalized Lipodystrophy
CGL2: Type 2 Congenital Generalized Lipodystrophy
CGL4: Type 4 Congenital Generalized Lipodystrophy
CSS: Cascading Style Sheets
FPLD: Familial Partial Lipodystrophy
FPLD1: Type 1 Familial Partial Lipodystrophy.
FPLD2: Type 2 Familial Partial Lipodystrophy
FPLD4: Type 4 Familial Partial Lipodystrophy
FPLD5: Type 5 Familial Partial Lipodystrophy
FPLD6: Type 6 Familial Partial Lipodystrophy
IOS: iPhone Operative System
HGPS: Hutchinson-Gilford progeria syndrome
HIV: Human immunodeficiency viruses
HTML: HyperText Markup Language
MADA: Type A mandibuloacral dysplasia
MDPL: Mandibular hypoplasia, deafness, progeroid features, and lipodystrophy syndrome
MIT: Massachusetts Institute of Technology
OMIM: Online Mendelian Inheritance in Man
PELD: Progressive Encephalopathy with/without Lipodystrophy
PRAAS: Proteasome-associated auto-inflammatory syndrome
SCSS: Sassy CSS
SHORT: Short stature, hyperextensibility, hernia, ocular depression, Rieger anomaly, and teething delay
SPSS: Statistical Package for the Social Sciences
