# Supplementary material for: LipoDDx: a mobile application for identification of rare lipodystrophy syndromes
Source: Orphanet J Rare Dis. 2020 Apr 2;15:81. doi: 10.1186/s13023-020-01364-1 (PMC7118879; doi:10.1186/s13023-020-01364-1)
Supplement: Supplementary file 1 — Additional file 1: Table 1S. Score table. The result obtained by a particular evaluator for a particular case will receive different score according to the difficulty of the diagnosis. Because the degree of difficulty in the diagnosis is not equal in all of the cases, we assigned a different score, ranged between -10 to 40, according to the “difficulty” for diagnosing. For instance, if a patient has a loss of fat because she had anorexia nervosa but the evaluator considered that she suffered any subtype of generalized lipodystrophy, the score will be negative, and the opposite is true, if a patient suffered a extremely infrequent lipodystrophy, with a complicated phenotype (for instance, Wiedemann Rautenstrauch syndrome) and the evaluator achieved a correct diagnose, the score will be high. Then, we add all the scores of each evaluator , transformed in percentage, and compared the results obtained based on his/her on knowledges with those provided by LipoDDX. [file 13023_2020_1364_MOESM1_ESM.pdf]

Table 1S. Score for validation

Evaluator diagnosis	FPLD2	AGL	APL	FPLD6	Keppen-Lubinsky	FPLD3	TRANSPLANT	PELD	CGL2	CGL1	MARFAN	PRAAS1	SHORT
**Correct diagnosis**													
**FPLD2**	20	0	0	10	-5	15	15	0	0	0	-5	-5	0
**AGL**		20	0	0	-5	0	0	0	15	15	-5	-5	0
**APL**			20	0	-5	0	0	0	0	0	-5	-5	10
**FPLD6**				20	-5	15	15	0	0	0	-5	-5	-5
**Keppen-Lubinsky**					40	0	0	0	0	0	0	0	0
**FPLD3**						20	15	0	0	0	-5	-5	-5
**TRANSPLANT**							20	0	0	0	-5	-5	-5
**PELD**								30	20	20	-5	-5	-5
**CGL2**									20	20	-5	-5	-5
**CGL1**										20	-5	-5	-5
**MARFAN**											35	0	0
**PRAAS1**												30	0
**SHORT S.**													40

Table 1S. Score for validation

Evaluator diagnosis	MDPL	No Lipodystrophy	WERNER	FPLD4	Ruijs-Aalfs S.	Localized	Cockayne	FPLD5	MFN2	CGL4	Nestor-Guillermo P..S.	Atypical Prog.S.
**Correct diagnosis**												
**FPLD2**	-5	-10	-5	15	-5	-5	-5	15	15	0	-5	-5
**AGL**	-5	-10	-5	0	-5	-5	-5	0	0	15	-5	-5
**APL**	-5	-10	-5	0	-5	-5	-5	0	0	0	-5	-5
**FPLD6**	-5	-10	-5	15	-5	-5	-5	15	15	0	-5	-5
**Keppen-Lubinsky**	0	-10	0	0	0	-5	0	0	0	0	0	0
**FPLD3**	-5	-10	-5	15	-5	-5	-5	15	10	0	-5	-5
**TRANSPLANT**	-5	-10	-5	15	-5	-5	-5	15	10	0	-5	-5
**PELD**	-5	-10	-5	0	-5	-5	-5	0	0	0	-5	-5
**CGL2**	-5	-10	-5	0	-5	-5	-5	0	-5	10	-5	-5
**CGL1**	-5	-10	-5	0	-5	-5	-5	0	0	15	-5	-5
**MARFAN**	0	-10	0	0	0	-5	-5	0	0	0	0	0
**PRAAS1**	0	-10	0	0	0	-5	0	0	0	0	0	0
**SHORT**	0	-10	0	0	0	-5	0	0	0	0	0	0

Table 1S. Score for validation

Evaluator diagnosis	HGPS	ADRA2A	Fontaine PS	MADA	Wiedemann	FPLD1
**Correct diagnosis**						
**FPLD2**	-5	15	-5	-5	-5	15
**AGL**	-5	0	-5	-5	-5	0
**APL**	-5	0	-5	-5	-5	0
**FPLD6**	-5	15	-5	-5	-5	15
**Keppen-Lubinsky S.**	0	0	0	0	0	0
**FPLD3**	-5	15	-5	-5	-5	15
**TRANSPLANT**	-5	15	-5	-5	-5	15
**PELD**	-5	0	-5	-5	-5	0
**CGL2**	-5	0	-5	-5	-5	0
**CGL1**	-5	0	-5	-5	-5	0
**MARFAN**	0	0	0	0	0	0
**PRAAS1**	0	0	0	0	0	0
**SHORT**	0	0	0	0	0	0

Table 1S. Score for validation

Evaluator diagnosis	MDPL	No Lipodystrophy	WERNER	FPLD4	Ruijs-Aalfs S.	Localized	Cockayne	FPLD5	MFN2	CGL4	Nestor-Guillermo P..S.	Atypical Prog.S.
**Correct diagnosis**												
**MDPL**	35	-10	0	0	0	-5	0	0	0	0	0	0
**No Lipodystrophy**		1	-10	-10	-10	-10	-10	-10	-10	-10	-10	-10
**WERNER S.**			30	0	0	-5	0	0	0	0	0	0
**FPLD4**				20	-5	-5	-5	15	15	0	-5	-5
**Ruijs-Aalfs S.**					40	-5	0	0	0	0	0	0
**Localized**						1	-5	-5	-5	-5	-5	-5
**Cockayne**							40	0	0	0	0	0
**FPLD5**								20	15	0	-5	-5
**MFN2**									30	0	-5	-5
**CGL4**										25	-5	-5
**Nestor-Guillermo P..S.**											40	0
**Atypical Prog.S.**												30

Table 1S. Score for validation

Evaluator diagnosis	HGPS	ADRA2A	Fontaine PS	MADA	Wiedemann	FPLD1
**Correct diagnosis**						
**MDPL**	0	0	0	0	0	0
**No Lipodystrophy**	-10	-10	-10	-10	-10	-10
**WERNER**	0	0	0	0	0	0
**FPLD4**	-5	15	-5	-5	-5	15
**Ruijs-Aalfs S.**	0	0	0	0	0	0
**Localized Lipodystrophy**	-5	-5	-5	-5	-5	-5
**Cockayne S.**	0	0	0	0	0	0
**FPLD5**	-5	15	-5	-5	-5	15
**MFN2**	-5	15	-5	-5	-5	10
**CGL4**	-5	-5	-5	-5	-5	0
**Nestor-Guillermo P..S.**	30	0	0	10	0	0
**Atypical Prog.S.**	5	0	0	5	0	0

Table 1S. Score for validation

Evaluator diagnosis	HGPS	ADRA2A	Fontaine PS	MADA	Wiedemann	FPLD1	
**Correct diagnosis**							
**HGPS**	30	0	0	5	0	0	
**ADRA2A**		20	-5	-5	-5	15	
**Fontaine PS**			40	0	30	0	
**MADA**				30	0	0	
**Wiedemann**					40	0	
**FPLD1**						20	
							989

FPLD2: Type 2 Familial Partial Lipodystrophy; AGL:Acquired Generalized Lipodystrophy; APL : Acquired Partial Lipodystrophy; FPLD6: Type 6 Familial Partial Lipodystrophy; TRANSPLANT: Lipodystrophy associated with total body irradiation and hematopoietic stem cell transplant; PELD: Progressive Encephalopathy with/without Lipodystrophy; CGL2: Type 2 Congenital Generalized Lipodystrophy; CGL1 : Type 1 Congenital Generalized Lipodystrophy; MARFAN: Marfan syndrome with neonatal progeroid –like lipodystrophy; PRAAS1: Proteasome-associated auto-inflammatory syndrome; MDPL: mandibular hypoplasia, deafness, progeroid features, and lipodystrophy syndrome; FPLD4: Type 4 Familial Partial Lipodystrophy; FPLD5 : Type 5 Familial Partial Lipodystrophy; MFN2: *MFN2* associated FPLD; CGL4: Type 4 Congenital Generalized Lipodystrophy; Nestor-Guillermo P..S.: Nestor-Guillermo Progeria Syndrome; Atypical Prog.S.: Atypical Progeria Syndrome; HGPS: Hutchinson-Gilford progeria syndrome; ADRA2A: *ADRA2A* associated FPLD; Fontaine PS: Fontaine progeroid syndrome; MADA: Type A mandibuloacral dysplasia; Wiedemann: Wiedemann Rautenstrauch syndrome; FPLD1: Type 1 Familial Partial Lipodystrophy.
